# Unexpected associated microalgal diversity in the lichen *Ramalina farinacea* is uncovered by pyrosequencing analyses

**DOI:** 10.1371/journal.pone.0175091

**Published:** 2017-04-14

**Authors:** Patricia Moya, Arántzazu Molins, Fernando Martínez-Alberola, Lucia Muggia, Eva Barreno

**Affiliations:** 1 Dpto. Botánica, Instituto Cavanilles de Biodiversidad y Biología Evolutiva, Fac. CC. Biológicas, Universitat de València, Burjassot, Valencia, Spain; 2 University of Trieste, Department of Life Sciences, Trieste, Italy; Wilfrid Laurier University, CANADA

## Abstract

The current literature reveals that the intrathalline coexistence of multiple microalgal taxa in lichens is more common than previously thought, and additional complexity is supported by the coexistence of bacteria and basidiomycete yeasts in lichen thalli. This replaces the old paradigm that lichen symbiosis occurs between a fungus and a single photobiont. The lichen *Ramalina farinacea* has proven to be a suitable model to study the multiplicity of microalgae in lichen thalli due to the constant coexistence of *Trebouxia* sp. TR9 and *T*. *jamesii* in long-distance populations. To date, studies involving phycobiont diversity within entire thalli are based on Sanger sequencing, but this method seems to underestimate the diversity. Here, we aim to analyze both the microalgal diversity and its community structure in a single thallus of the lichen *R*. *farinacea* by applying a 454 pyrosequencing approach coupled with a careful *ad hoc*-performed protocol for lichen sample processing prior to DNA extraction. To ascertain the reliability of the pyrosequencing results and the applied bioinformatics pipeline results, the thalli were divided into three sections (apical, middle and basal zones), and a mock community sample was used. The developed methodology allowed 40448 filtered algal reads to be obtained from a single lichen thallus, which encompassed 31 OTUs representative of different microalgae genera. In addition to corroborating the coexistence of the two *Trebouxia* sp. TR9 and *T*. *jamesii* taxa in the same thallus, this study showed a much higher microalgal diversity associated with the lichen. Along the thallus ramifications, we also detected variations in phycobiont distribution that might correlate with different microenvironmental conditions. These results highlight *R*. *farinacea* as a suitable material for studying microalgal diversity and further strengthen the concept of lichens as multispecies microecosystems. Future analyses will be relevant to ecophysiological and evolutionary studies to understand the roles of the multiple photobionts in lichen symbioses.

## Introduction

Lichens have been traditionally described as symbiotic organisms (holobionts) resulting from the close morpho-physiological interaction between a heterotroph (mycobiont) and at least one photosynthetic (photobiont) partner, which can be green microalga (phycobiont) and/or cyanobacteria (cyanobiont) [1]. Recently, additional complexity was reported inside a single lichen thallus by the intrathalline coexistence of multiple microalgae [2–7], bacteria and yeasts [8,9], overthrowing the old paradigm that lichens considered to be mutualistic associations between one lichenized fungus and a single photobiont.

Previous studies performed on the lichen *Ramalina farinacea* (L.) Ach. showed the recurrent co-occurrence of at least two phycobionts (*Trebouxia* sp. TR9 and *Trebouxia* sp. TR1 *= T*. *jamesii*) inside the thalli using microscopic techniques, culture isolations of both phycobionts and molecular characterization with different genetic markers [10,11]. The coexistence of these two phycobionts was verified in several populations of *R*. *farinacea* from the Iberian Peninsula, the Canary Islands and North America [3,11]. Moreover, several studies have demonstrated that these two phycobionts respond differently to abiotic stress [3,12–14]; therefore, their coexistence would be advantageous for the whole lichen under extreme environmental conditions and would represent a common, basic phenomenon in ecologically adapted lichens.

Recently, deep sequencing techniques have been increasingly used to analyze the diversity of lichen-associated bacteria [11,15–17] and fungi, such as lichenicolous and endolichenic fungi [18–22] and, to a lesser extent, microalgal communities [19,22]. Although 454 pyrosequencing has been already surpassed by other NGS approaches, it still represents a powerful and complementary approach to the traditional Sanger sequencing or fingerprinting techniques for biodiversity assessments. Several authors have also uncovered the promiscuity of phycobionts associated with lichen thalli using analyses of conformation polymorphism of the ITS fragments [11,23], microsatellites [24], and microscopic examinations [6,25]. Because high-throughput sequencing (HTS) allows the detection of a vast number of genotypes, which otherwise remain underestimated with conventional PCR amplifications [26], meta-community studies represent a potential tool to address questions about lichen phycobiont populations and their interactions with the surrounding environments [27].

Here, we applied the 454 pyrosequencing approach, complementing traditional Sanger sequencing, to considerably improve our knowledge of the microalgal diversity associated with a lichen thallus, using *R*. *farinacea* as a model system. We designed a reliable protocol for the preparation of lichen samples prior to DNA extraction and took particular care to differentiate the most basal and the most apical parts of the laciniae (thallus ramifications) in order to detect any possible differential in community structure. In particular, we aimed at answering the following questions: i) Does *R*. *farinacea* host other phycobionts in addition to the already co-occurring *Trebouxia* sp. TR9 and *T*. *jamesii*? ii) If additional microalgae are present, how diverse are they? iii) Are the co-occurring phycobionts differentially localized along the laciniae? and iv) To what extent does a 454 pyrosequencing approach complement the results obtained by traditional Sanger sequencing?

## Materials and methods

### Sampling and experimental design

**Fig 1:**
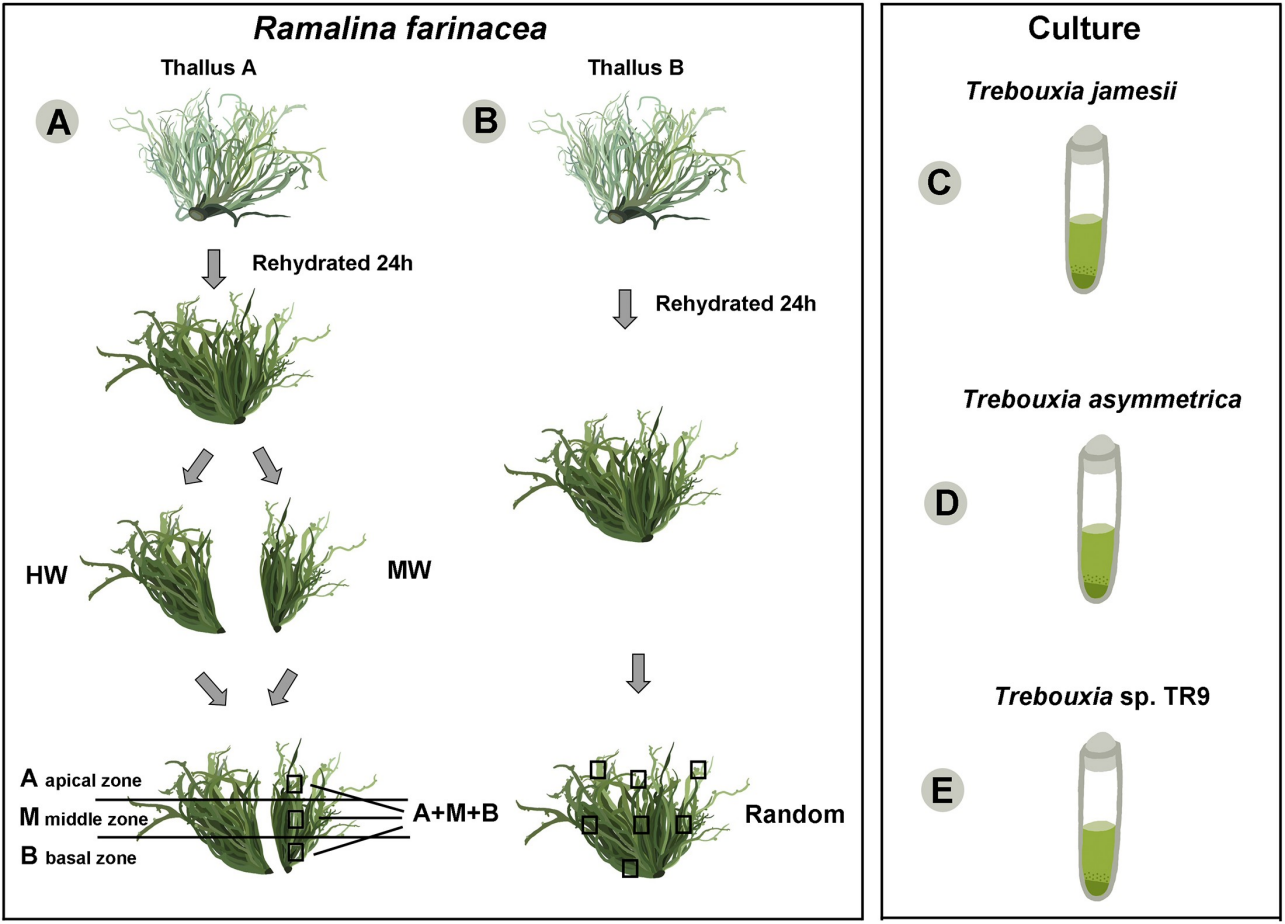
Preparation of lichen material prior to DNA extraction performed in this study. Two thalli of *Ramalina farinacea* (thallus A and B) were first rehydrated for 24 h. Thallus **A** was divided at the base into two halves: the first was washed with sterile water (HW), and the second was treated as described by Muggia et al. [23] (MW). The two halves were paralleled and divided into three sections: an apical zone (A), a middle zone (M) and a basal zone (B). One part of each section was selected and mixed (A+M+B). The second thallus **B** was washed as in the HW treatment; several parts of this thallus were randomly selected and mixed (Random). The template DNA for further analysis was extracted from the HW, MW, A, M, B, A+M+B and Random treatments. Algal cultures used as controls: **C**, *Trebouxia jamesii*, **D**, *T*. *asymmetrica* and **E**, *Trebouxia* sp. TR9.

*Ramalina farinacea* is usually an epiphytic, pendant, fruticose lichen that is attached to the substratum by a central, basal hapter and presents abundant powdery outbreaks (soralia) containing asexual dispersal propagules (soredia). The lichen grows on a wide variety of substrata (phorophytes) and in diverse habitats [28]. Two specimens of *R*. *farinacea* were collected for this study from one locality (Spain, Tenerife, Canary Island, Los Realejos, 28°20´38.34"N/16°34´40.30"W) (permits were not required). Samples were dried and stored at -20°C until processing. Lichen thalli were rehydrated with Milli-Q sterile water one day before being processed and stored in a growth chamber at 20°C under a 12 h/12 h light/dark cycle (lighting conditions: 15 μmol/m^2^/s). Lichen thalli were examined under a stereomicroscope to remove surface contamination (e.g., bark, sand, mosses, fragments of other lichen species, or infection by lichenicolous fungi). The first thallus was divided into two halves (Fig 1A). The first half was vortexed 3 times for 5 min at 2000 rpm with Milli-Q sterile water; this water was kept and labeled HW (H_2_O washing). The second half was washed as described by Muggia et al. [23] using a 1% solution of Tween80 in distilled sterile water; this washing water was also kept and labeled MW (Muggia washing).

The two thallus halves were paralleled and divided into three sections that were individually treated further (Fig 1A): an apical zone (A), a middle zone (M) and a basal zone (B). The fragments coming from the three different sections were pooled together, and this pooled sample was labeled ‘A+M+B’. The second thallus (Fig 1B) was washed following the same procedure that was performed with HW; alternatively, the thallus parts were randomly excised and pooled together, and this sample was named ‘Random’.

The reliability of the bioinformatics pipelines and the sensitivity of the technique employed in this study was verified by pyrosequencing of the two *Trebouxia* species, *T*. *jamesii* SAG 2103 (Culture Collection of Algae at Goettingen University) and *T*. *asymmetrica* SAG 48.88, which was retrieved from SAG. The *Trebouxia* sp. TR9 was available in our culture collection at the Universitat de València (Fig 1C–1E). We also included as control an *in vitro* mock community composed of an equimolar mix of DNA of *T*. *jamesii* and *T*. *asymmetrica* culture isolates.

### DNA extraction, PCR and sanger sequencing

The total genomic DNA was extracted from the HW, MW, A, M, B, A+M+B, and Random treatments and from the cultures of *T*. *jamesii*, *T*. *asymmetrica* and *Trebouxia* sp. TR9 using the DNeasy Plant Mini kit (Qiagen, Hilden, Germany) following the manufacturer’s instructions.

The fungal nrITS DNA was analyzed to confirm the identity of the mycobiont from the two lichen thalli used. The fungal nrITS DNA was amplified using the primer pair ITS1F [29] and ITS4 [30].

The algal locus encoding the nrITS DNA was amplified using the primer pair nr-SSU-1780 [31] and 5.8S 2R designed in our laboratory (5´-CGT TCA AAG ATT CGA TGG-3´) based on the ITS1-5.8S alignment of *Trebouxia*, *Asterochloris*, *Coccomyxa* and *Dictyochloropsis*. The PCRs were performed in 50 μl using EmeraldAmp GT PCR Master Mix (Takara, Shiga, Japan), which required the addition of the template DNA, specific primers and water, which allowed for improved reproducibility while minimizing the potential for contamination. The PCR program for amplification comprised an initial denaturation at 94°C for 2 min and 30 cycles at 94°C for 30 s, 56°C for 45 s and 72°C for 1 min, followed by a final elongation at 72°C for 5 min. The amplifications were carried out on a 96-well SensoQuest labcycler (Progen Scientific Ltd., South Yorkshire, UK). The PCR products were visualized on 2% agarose gels and purified using the Gel Band Purification Kit (GE Healthcare Life Science, Buckinghamshire, England), after which they were quantified spectrophotometrically (Nanodrop 2000, Thermo Scientific, Wilmington, DE, USA). The amplified PCR products were sequenced with an ABI 3730XL Genetic Analyzer using the BigDye Terminator v 3.1 Cycle Sequencing Reaction Kit (Applied Biosystems, Foster City, CA).

### Real Time-PCR (RT-PCR) and PCR for 454 pyrosequencing

**Fig 2:**
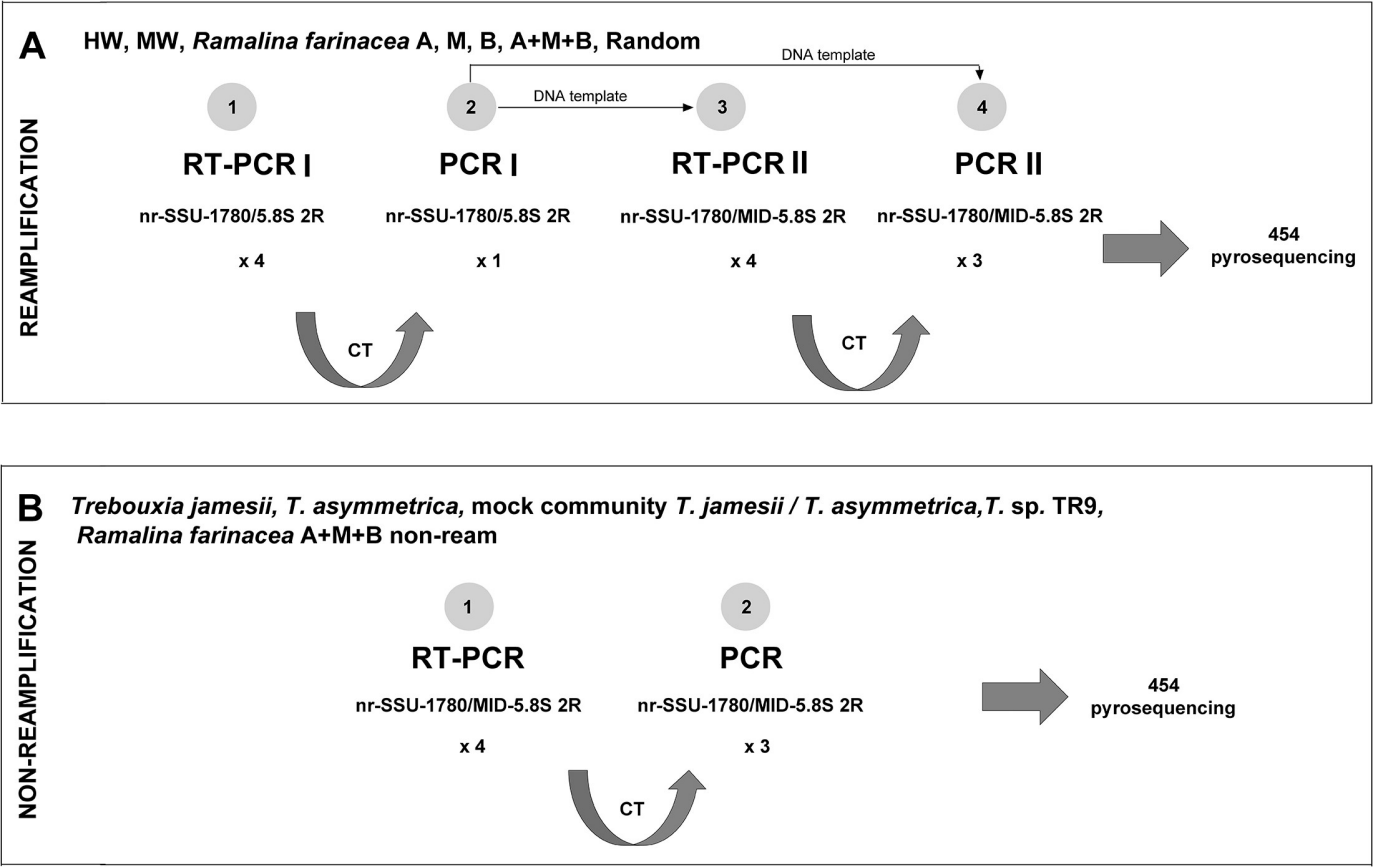
Scheme of Real Time-PCR (RT-PCR) strategies. For the preparation of the algal DNA, two strategies were followed: **A** a ‘reamplification strategy’ for the HW, MW, A, M, B, A+M+B and Random treatments and **B** a ‘non-reamplification strategy’ for samples of the individual *T*. *jamesii*, *T*. *asymmetrica* and *Trebouxia* sp. TR9 cultures, the mock community, and the A+M+B non-ream. See the text for more details. CT: cycle threshold.

The preparation of the algal DNA for pyrosequencing followed two strategies (Fig 2). We performed a ‘reamplification strategy’ for the HW, MW, A, M, B, A+M+B and Random treatments (Fig 2A) and a ‘non-reamplification strategy’ (‘non-ream’) for samples of the individual *T*. *jamesii*, *T*. *asymmetrica* and *Trebouxia* sp. TR9 cultures and the mock community and for the A+M+B treatment, which were distinguished as ‘A+M+B non-ream’ (Fig 2B).

In the reamplification strategy (Fig 2A) an initial RT-PCR (RT-PCR I) and an initial PCR (PCR I) were performed using the genomic DNA as a template and the nr-SSU-1780/5.8S 2R primers. The number of cycles of PCR I were determined by the average Ct of the RT-PCR I (Fig 2A.1 and 2A.2). We then performed a second RT-PCR (RT-PCR II) and a second PCR (PCR II) using 1 μl of the PCR I product as template, and the fusion primers were designed following the GS Junior System Guidelines for Amplicon Experimental Design (Roche, Branford, USA), employing the unidirectional sequencing protocol for library construction (Lib-L chemistry for emulsion PCR, emPCR, ‘One-Way Reads’). The forward fusion primer was 5’-A-KEY-nr-SSU-1780–3’, and the reverse fusion primer was 5’-B-KEY-MID-5.8S-2R-3’, where A and B represent the pyrosequencing adaptors; the multiplex identifier (MID) was added for post-sequencing sample identification and was treatment-specific. The specific cycle number for PCR II was determined by the average Ct from the RT-PCR II (Fig 2A.3 and 2A.4).

In the non-reamplification strategy (Fig 2B), only one RT-PCR and one PCR were performed using the genomic DNA as a template and the fusion primers as previously described. The number of cycles of the PCR was determined by the average Ct of the RT-PCR (Fig 2B.1 and 2B.2).

All RT-PCRs (20 μl) contained 10 μl of SYBR premix ExTaq (Takara, Shiga, Japan), 0.8 μM nr-SSU-1780/5.8S 2R primers, 0.4 μl of ROX reference, and 1 μl of template DNA, and sterile Milli-Q water was used to bring to volume. Each run contained quadruplicate samples using the following thermal cycle conditions: 30 s at 95°C followed by 40 cycles of 5 s at 95°C and 30 s at 60°C. In this analysis, the ABI StepOnePlus (Applied Biosystems, Foster City, CA, USA) was used. The Ct values were determined using the StepOne software v 2.1 package (Applied Biosystems) based on the fluorescence data recorded during each RT-PCR run. All PCRs (25 μl) contained 2.5 μl of 10X buffer, 0.4 μM nr-SSU-1780/5.8S 2R primers, 0.2 mM dNTPs, and 0.6 u/μl of ExTaq (Takara, Shiga, Japan), and sterile Milli-Q water was used to bring to volume. The PCR conditions were 1 cycle of 95°C for 2 min; X number of cycles (S1 Table) of 94°C for 30 s, 56°C for 45 s, and 72°C for 1 min; and a final extension of 72°C for 5 min.

### Library preparation

PCRs (Fig 2A.4 and 2B.2) were performed in triplicate following the previously described methodology to prepare the amplicon generation libraries. The amplicons were double purified using the Agencourt AMPure XP Bead PCR Purification protocol (Beckman Coulter Genomics, MA, USA). After purification, the amplicons were visualized in a Bioanalyzer 2100 and quantified fluorometrically using the Quant-iT PicoGreen kit (Invitrogen Molecular Probes, Eugene, OR, USA). The three PCR products from each sample were pooled together based on their concentration. The amplicon libraries were combined in a single tube in equimolar concentrations. The pooled amplicon mixture was purified twice (AMPure XP kit, Agencourt, Takeley, United Kingdom), and the cleaned pool was quantified again using the PicoGreen assay (Quant-iT, PicoGreen DNA assay, Invitrogen).

Subsequently, the amplicons were submitted to the pyrosequencing service Lifesequencing S.L. (Valencia, Spain), where an emulsion PCR was performed, and unidirectional pyrosequencing was carried out on a GS Junior 454 system (Roche 454 Life Sciences, Branford, CT, USA) following the Roche Amplicon Lib-L protocol.

### Data analysis

The sequences were sorted into separate files according to their treatment-specific multiplex identifier using the sff script file included in the Roche Newbler package (http://www.454.com/products/analysis-software/). The amplicon sequences from each SFF file were extracted using the sff_extract script (http://bioinf.comav.upv.es/sff_extract/index.html) to generate fasta and quality files that were combined into a single fastq file with a custom Python script. The fastq file was examined with FastQC software v 0.10.1 [32] to inspect the length and quality of the reads. Processing and trimming of the low-quality ends of the sequences were performed with the program Trim_Edges from the seq_crumbs-0.1.8 package based on the graphs generated with the FastQC software. Eighteen nucleotides from 5’-ends (sequencing primer and MID sequences) and 22 nucleotides from 3’-ends (low-quality nucleotides) were removed. Sequences shorter than the final sequence alignment were not considered in the analyses. BLASTCLUST software v 2.2.26 [33] was employed using different score coverage threshold values to test for operational taxonomic units (OTUs) clustering with a custom-curated database including 22 sequences from the SAG, UTEX (Culture Collection of Algae at the University of Texas) and reference sequence of *Trebouxia* sp. TR9. The resulting fasta file from each library was clustered based on a 99% score coverage threshold and a 90% length coverage threshold (S 99-L 0.9). Each OTU obtained from the output clustering list file was converted into an individual fasta file using a custom Python script that included the removal of unique sequences (singletons). The different sequences from each OTU were aligned by MUSCLE [34] and manually adjusted using MEGA v 5.0 [35], and a consensus sequence file was generated for each OTU. In subsequent analyses, these consensus sequences were encoded as treatment code_number of clustering group_number of sequences and were classified into genera (*Trebouxia*, *Asterochloris* or any of the additional algal genera) using BLAST searches [33] against the GenBank sequence database with the entry query filter “UTEX OR SAG”. All sequences obtained in this study have been submitted to NCBI/SRA under the bioproject identification number PRJNA82781.

### Phylogenetic analysis

An initial multiple alignment was prepared including the i) newly determined *Trebouxia* consensus ITS1-5.8S sequences, ii) 22 nrITS DNA of well-accepted *Trebouxia* species available from the SAG and UTEX, iii) *Trebouxia* sp. TR9 (KU716051) sequence and iv) representative ITS sequences of 51 OTUs described by Leavitt et al. [7] (downloaded from the Dryad repository: Dryad doi:10.5061/dryad.5rm6d).

A second multiple alignment was prepared for sequencing matching of *Asterochloris*. It included the newly determined consensus partial ITS1-5.8S sequences and 14 nrITS DNA for representative *Asterochloris* species described by Škaloud et al. [36] available from the UTEX, SAG, Culture Collection of Algae at the University of Prague (CAUP), and Culture Collection of Algae and Protozoa (CCAP) as well as the *A*. *mediterranea* (KP257366) sequence downloaded from GenBank.

The *Trebouxia* and *Asterochloris* datasets were individually aligned by MUSCLE and manually adjusted using MEGA v 5.0. Both the phylogenetic relationships and their confidence values were inferred using maximum likelihood (ML) implemented in RAxML v 8.0.5 [37]. All ML searches followed a GTRGAMMA model of molecular evolution, and support values for the topology were calculated using a 1000-bootstrap approach. Tree files were midpoint rooted with the program FigTree (http://tree.bio.ed.ac.uk/software/figtr4), and features were added with the graphical software GIMP v 2.8.10 (http://www.gimp.org/) and Inkscape v 0.48.4 r9939 (https://inkscape.org/en/).

The genetic diversity among sequences obtained for *Trebouxia* was further analyzed by haplotype networks using TCS software [38] on the basis of maximum parsimony analyses. We included in the haplotype network all sequences used in the phylogenetic analysis.

We used the automatic barcode gap discovery (ABGD) [39] analysis to circumscribe OTUs representing candidate species following the parameters for *Trebouxia* spp. used by Leavitt et al. [7] and applying the default parameters for the additional green microalgae OTUs. The ABGD program employs a genetic distance-based approach to detect OTUs representing candidate species.

BLAST searches were performed to identify the chlorophyte sequences up to, at least the genus level.

### Specific primer design

We designed specific forward primers (S2 Table) to detect the different OTUs recovered from the pyrosequencing analysis by PCR using the DNA template originally extracted from the seven treatments. Semi-nested PCR was performed first with nr-SSU-1780/5.8S 2R followed by a nested reaction with the newly developed specific internal primers and reverse primer 5.8S 2R. The PCR conditions were as described above.

### Rank abundance and diversity index

Rank abundance curves were constructed to visualize the relative biological diversity structure, richness and evenness (abundance distribution of species in a community) of *Trebouxia*, *Asterochloris* and the additional green microalgae.

The OTU diversity found in each treatment was calculated by the Margalef (D_mg_), the Shannon (H´) and the Simpson (D´) diversity indices according to the methods of LexerØd [40] and Samo [41].

## Results

### PCR amplification and sanger sequencing

The identity of the mycobiont *Ramalina farinacea* was confirmed by BLAST analyses against the GenBank database for A, M, B, A+M+B and Random as well as the two washing treatments (HW and MW; Fig 1).

The identity of the algal cultures and that of the primary phycobiont present in the individual HW, MW, A, M, B, A+M+B and Random treatments was confirmed by BLAST searches of the nrITS DNA sequences. Significant matches of 95%-99% identity and 97%-100% coverage were obtained (S1 Table). Only the B and HW treatments resulted in double bands in the agarose gel. These two bands were individually purified from the gel and sequenced. They corresponded to *Trebouxia* sp. TR9 and *Asterochloris mediterranea* (KT215311) in both the B and HW treatments (S1 Table).

### Real-Time PCR (RT-PCR)

The over-amplification of the primary phycobiont was circumvented by performing RT-PCR. Rather than using a fixed PCR cycle number for all samples, the appropriate PCR cycle number for the 454 pyrosequencing assay was set based on the cycle threshold (Ct). Re-amplified samples showed Ct values ranging from 20 to 29 for the initial PCR I round and 5 to 10 in PCR II (S2 Table); the non-reamplified samples presented Ct values from 20 to 30 (S2 Table).

### Sequencing throughput and quality control of 454 pyrosequencing

Two sequencing runs were performed in this study. The first run was performed on a plate comprising 24 multiplex identifiers (MIDs) including the three algal cultures, the *in vitro* mock community (equimolar mix of DNA of *T*. *jamesii* and *T*. *asymmetrica*) and the amplifications of the seven treatments obtained from thallus A (HW, MW, A, M, B, A+M+B, and A+M+B non-ream). The second run was performed on a plate comprising 82 MIDs, which included the Random treatment from thallus B only. Raw read datasets obtained from the cultures and the eight treatments were individually trimmed, and singletons and unreliable reads were filtered and removed.

The filtered dataset for the three algal cultures and the *in vitro* mock community resulted in 24485 reads (Table 1). The cleaned dataset of reads was reduced to less than 1% of the original number of reads. BLAST searches confirmed the individual identity of the *Trebouxia* species of the cultures and of the two species used for the mock community (86% of sequences for *T*. *jamesii* and 14% for *T*. *asymmetrica*).

**Table 1:** Summary of the pyrosequencing results performed on the individual algal cultures, the mock community and the eight treatments.

MIDs	Raw reads	Filtered reads	Singletons/ Unreliable Reads	N. of *Trebouxia* OTUs	Presence of *Asterochloris* OTUs	N. of additional green microalgae OTUs
***T*. *jamesii***	6932	6855	44/33	1	No	0
***T*. *asymmetrica***	4446	4427	19/0	1	No	0
**Mock community (*T*. *jamesii/T*. *asymmetrica*)**	6970	6935	35/0	2	No	0
***Trebouxia* sp. TR9**	6309	6268	41/0	1	No	0
**HW**	4260	4191	69/0	5	Yes	2
**MW**	4741	4683	58/0	5	Yes	0
**A**	6333	6192	105/19	18	Yes	5
**M**	6613	6462	89/57	16	Yes	2
**B**	7017	6848	122/47	18	Yes	4
**A+M+B**	4947	4910	37/0	5	Yes	0
**A+M+B non-ream**	7274	7162	97/15	13	Yes	5
**Random**	1532	1493	39/0	10	No	0

A total clean dataset of 40448 reads was obtained for the seven amplifications from thallus A summed together, and this dataset was considered for the further analyses (Table 1).

The Random treatment from thallus B resulted in a clean dataset of 1493 total reads. The total dataset from the two sequencing runs comprised 66426 good-quality reads (Table 1).

### Clustering score coverage threshold analysis

A representative reference ITS1-5.8S sequence database comprising 22 well-accepted *Trebouxia* species from the UTEX and SAG and *Trebouxia* sp. TR9 were used to test the optimal threshold clustering value. A fasta file containing these sequences was created and clustered at different threshold values. Clustering at 100% and at 99% separated the highest numbers of *Trebouxia* species (23). Hence, for all of the analyses, the 99% score coverage threshold was used for OTU clustering in an attempt to reduce the potential increase of singletons when applying a 100% coverage threshold due to the presence of sequencing homopolymer errors [42].

### *Trebouxia*, *Asterochloris* and additional green microalgae diversity

**Fig 3:**
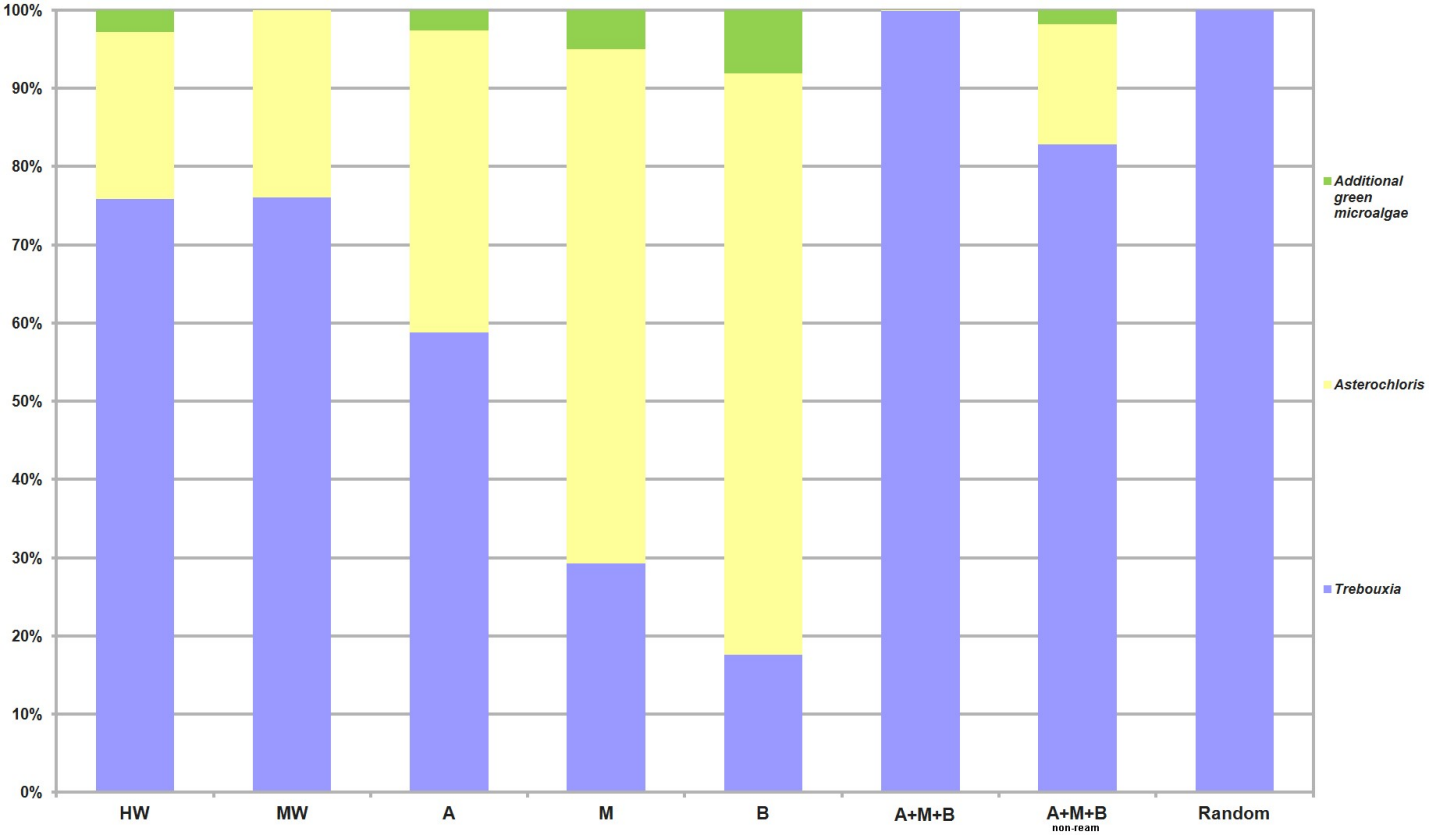
Relative abundance plot of algal diversity recovered for each treatment. Relative abundance was calculated as the percentage of sequences belonging to *Trebouxia*, *Asterochloris* and additional green microalgae among all sequences recovered from each treatment. The color coding for each is reported on the right side.

The taxonomic abundance plot of the whole algal composition (Fig 3) shows that *Trebouxia* OTUs were present in all of the treatments. Only in M and B did *Trebouxia* OTUs represent less than 50% (29% and 17%, respectively). *Asterochloris* was the second-most dominant algal genus, being absent only in Random. The OTU representatives of other, multiple genera, OTU1-OTU8 (named “additional green microalgae”), were absent in the MW, A+M+B and Random treatments (Table 2).

**Table 2:** Taxonomic identification of the additional green microalgae according to BLAST matches in GenBank and numbers of the corresponding sequences present in each treatment. The number of OTUs estimated by the automatic barcode gap discovery (ABGD) approach are reported.

ABGD OTUs	HW	MW	A	M	B	A+M+B	A+M+B non-ream	Random	BLAST match
**1**	0	0	0	0	0	0	10	0	-
**2**	89	0	0	0	0	0	0	0	*Elliptochloris* sp. MRL-2009 clone 459 (JF913706)
**3**	0	0	4	0	0	0	0	0	-
4	0	0	5	0	9	0	24	0	*Chlorophyta* sp. URa28, Tuerk51503(KF907701)
**5**	0	0	22	43	147	0	9	0	-
6	0	0	116	277	372	0	68	0	*Chlorophyta* sp. URa26, Tuerk 51502
7	29	0	0	0	36	0	0	0	*Vulcanochloris guanchorum* A104
8	0	0	8	0	0	0	17	0	*Diplosphaera chodati* UTEX 1177
**Total number of OTUs**	2	0	5	2	4	0	5	0	

In the A, M, B, A+M+B and A+M+B non-ream treatments, a total of 31 OTUs were detected from thallus A (23 *Trebouxia*, 1 *Asterochloris* and 7 additional green microalgae), and only 10 *Trebouxia* OTUs from thallus B in the Random treatment were detected (S3 Table). A total of 24 OTUs from thallus A were detected (18 *Trebouxia* and 6 additional green microalgae), excluding the OTUs recovered in the washing treatments (HW = 8 OTUs and MW = 6 OTUs) but maintaining *Trebouxia* sp. TR9 and *T*. *jamesii*. In the case of thallus B (Random), only 7 *Trebouxia* OTUs appeared. *Elliptochloris* was strictly considered an epithalline alga (only in HW).

#### Trebouxia

**Fig 4:**
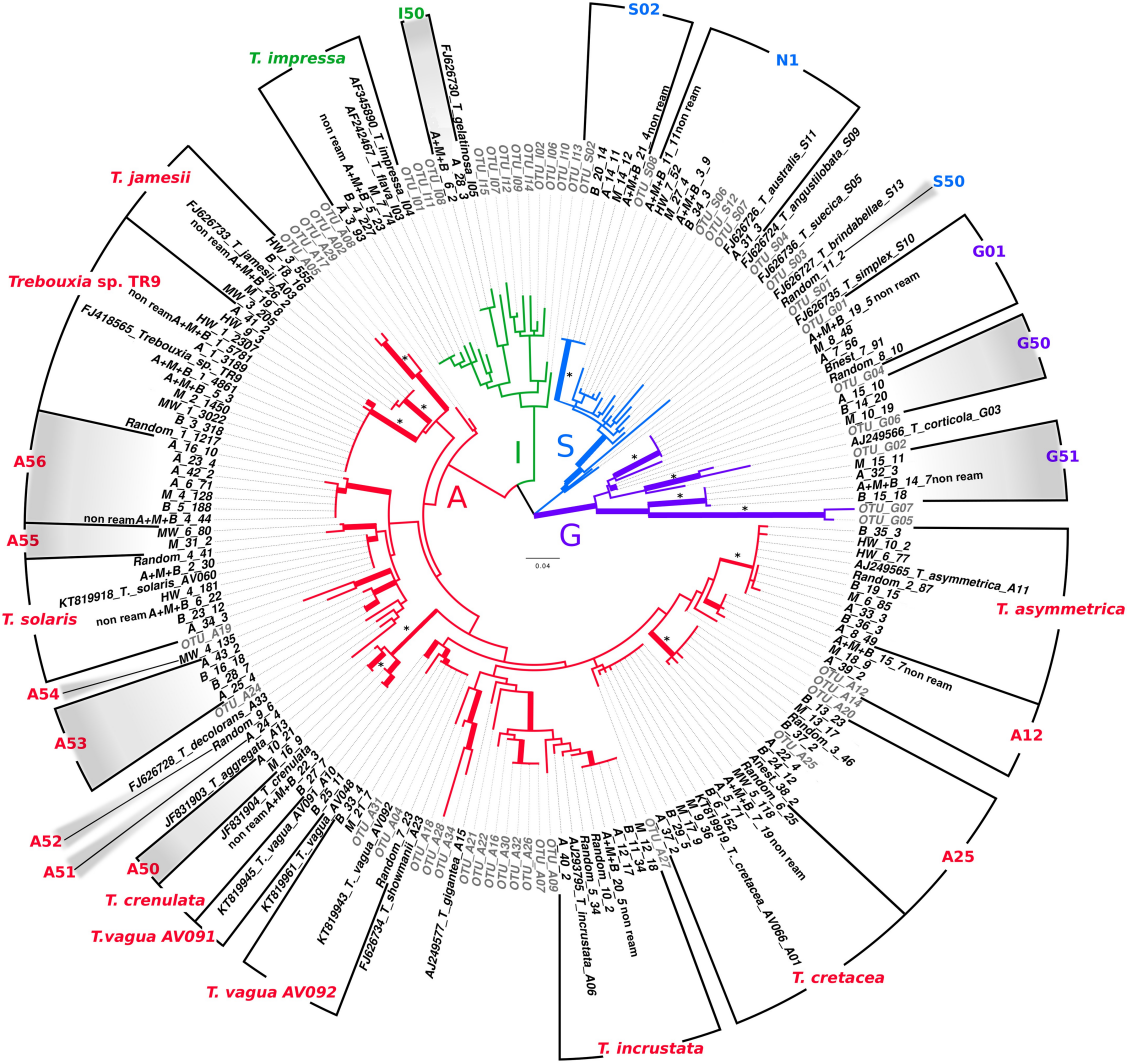
*Trebouxia* phylogenetic analysis. Midpoint rooted ITS1-5.8S gene tree representing 184 *Trebouxia* sequences, including 22 well-accepted *Trebouxia* species from the SAG and UTEX, *Trebouxia* sp. TR9 and 51 OTUs (in grey) described by Leavitt et al. [7] retrieved from GenBank. The pyrosequencing consensus sequences were encoded as treatment code_number of clustering group_number of sequences. Branches with an ML bootstrap support ≥ 75% are highlighted in bold, and branches with 100% bootstrap are additionally labeled by an asterisk (*). The four major clades described by Helms [44] are indicated (clades A, G, I, and S). Twenty-six *Trebouxia* OTUs detected in the pyrosequencing assay are indicated, and 11 unknown OTUs are also highlighted in grey.

The aligned algal ITS1-5.8S was 222 bp in length, excluding an InDel fragment (28 bp); this alignment presented 126 parsimony informative sites. The phylogenetic analysis was topologically congruent with those of previous studies [4,5,7,43] (Fig 4).

In the eight analyzed treatments, we detected a total of 26 OTUs corresponding to *Trebouxia*, of which nine correspond to well-accepted *Trebouxia* species and one to *Trebouxia* sp. TR9. Following the clade code of Helms [44], the majority of the OTUs belong to clade A–*arboricola*. The newly detected OTUs were named in the phylogenetic tree following the coding established by Leavitt et al. [7] and correspond to A50-A56 (Fig 4 and S3 Table).

Of the remaining eight *Trebouxia* OTUs, two were placed in clade I–*impressa*, including *T*. *impressa* and the new OTU I50; three *Trebouxia* were placed in clade G–*gigantea* (G01 and the new G50 and G51); and the other three *Trebouxia* in clade S–*simplex*. This last clade includes the OTUs S02 and S50 and the group N1, which remains partially unresolved and is represented here by OTUs S06, S07, and S12 as well as *T*. *australis* (Fig 4). Eleven OTUs of the 26 detected in *Trebouxia* were not previously detected by Sanger sequencing.

Both the haplotype parsimony networks (S1 Fig) and phylogenetic analyses (Fig 4) delimited 26 *Trebouxia* OTUs. However, 27 OTUs were defined using the ABGD analysis. This is because A_31_3, included in the N1 network, was considered in this network as a different OTU but was included in N1 clade in the phylogenetic analysis.

Intrathalline *Trebouxia*-specific PCR: To test for the presence of *Trebouxia* sp. TR9 and *T*. *jamesii*, specific primers previously designed by Casano et al. [3] were employed. For the other 24 pyrosequenced *Trebouxia* OTUs, we designed specific forward primers based on the nrITS DNA reference sequence obtained for each OTU (S4 Table). Only eight of them successfully amplified the targeted taxa *T*. *asymmetrica*, *T*. *crenulata*, *T*. *jamesii*, *T*. *solaris*, and *Trebouxia* sp. TR9 as well as OTUA25, OTUA52 and N1. The remaining specific primers that were designed only amplified *Trebouxia* sp. TR9 and therefore were not reported.

#### Asterochloris

**Fig 5:**
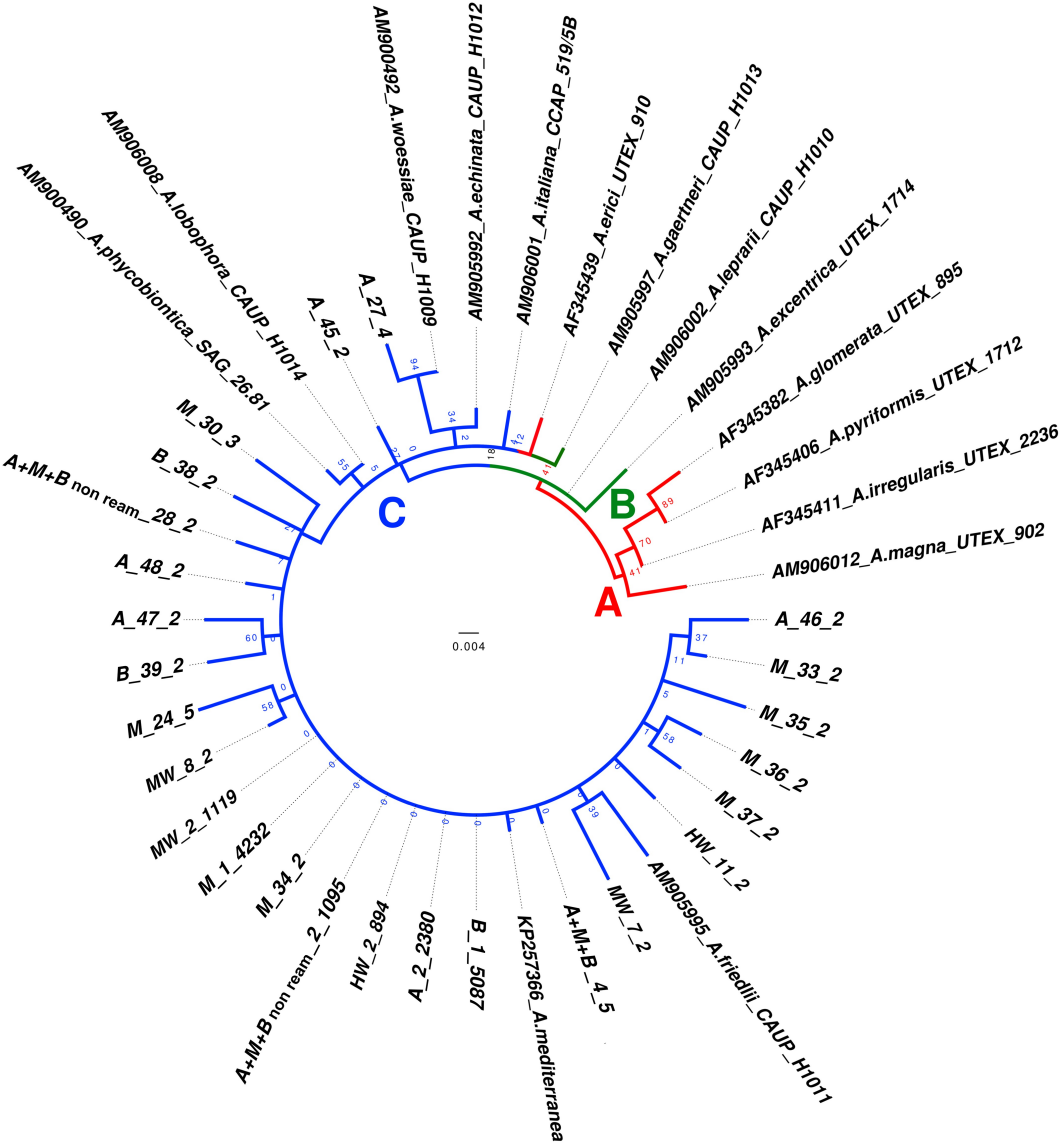
Phylogenetic analyses and haplotype analysis of *Asterochloris* taxa. **A**: Midpoint-rooted ITS1-5.8S gene tree representing 40 *Asterochloris* sequences, including 14 sequences selected from the SAG, UTEX, CAUP and CCAP and *A*. *mediterranea* retrieved from GenBank. Pyrosequencing consensus sequences were encoded as described in Fig 3. The values on the branches refer to ML bootstrap values. The three major clades described by Škaloud et al. [45] (2015) are indicated (clades A, B, and C).

*Asterochloris* sequences represented 35% the total sequences obtained in the pyrosequencing assay. The aligned algal ITS1-5.8S was 266 bp in length and included 5 parsimony informative sites. Our phylogenetic hypothesis is congruent with the previous analysis of Škaloud et al. [36], and the clades A, B and C were recovered. The only *Asterochloris* OTU (named *Asterochloris* sp.) circumscribed by phylogenetic analyses was placed in clade C (Fig 5). This *Asterochloris* OTU was not recovered in the Random treatment.

Intrathalline *Asterochloris*-specific PCR: Specific forward primers detected *Asterochloris* sp. in the seven different treatments (S4 Table).

#### Additional green microalgae

Sequences of additional green microalgal genera, representative of the genera *Elliptochloris*, *Vulcanochloris* and *Diplosphaera*, were obtained in 5 treatments: HW, A, M, B, and A+M+B non-ream (Table 2). The ABGD analysis identified 8 OTUs (OTU1-OTU8 in Fig 3 and Table 2) that produced BLAST matches of 90%-99% identity and 95%-100% coverage with five species—four species of Trebouxiophyceae and one species of Chlorophyceae. OTUs 1, 3 and 5 did not produce significant BLAST matches; therefore, we could not assign them a taxon name (Table 2).

OTU 2 (*Elliptochloris* sp.) was unique to HW, and OTU1 and OTU3 (unnamed) were unique to the A+M+B non-ream and A treatments, respectively.

Intrathalline additional green microalgae-specific PCR: We designed specific forward primers to amplify any of the eight new OTUs based on their nrITS DNA (S4 Table). Only one unknown OTU (AD_OTU1) was successfully amplified. The remaining specifically designed primers amplified either *Trebouxia* sp. TR9 or *A*. *mediterranea*, or they did not amplify anything and therefore were not reported.

### Biological diversity

Three indices (Margalef, Shannon and Simpson) were calculated for the eight treatments to evaluate and compare the diversity, richness and evenness of the algal communities. The Margalef index (Dmg = 0.71–3.21), the Shannon index (H*′* = 0.09–1.89) and the Simpson index (D´ = 1.02–2.72) indicated low diversity levels (Table 3).

**Table 3:** Diversity indices including *Trebouxia*, *Asterochloris* and the additional green microalgae groups detected in each treatment. B: Diversity indices only for *Trebouxia*.

	HW	MW	A	M	B	A+M+B	A+M+B non-ream	Random
Richness (S) =	8	6	24	19	23	5	19	10
Margalef Index (Dmg) =	1.03	0.71	3.21	2.27	2.58	2.49	2.57	1.23
Shannon Index (H´) =	1.89	1.49	1.72	1.61	1.70	0.09	0.99	1.19
Simpson Index (D´) =	2.72	2.10	2.42	2.06	1.78	1.02	1.48	1.49
Evenness (J´) =	0.63	0.58	0.37	0.37	0.38	0.04	0.23	0.36

HW (0.63) and MW (0.58) were the treatments with higher evenness, leading to higher diversity indices with low OTU richness. In contrast, in A+M+B (0.04) the OTU richness and evenness were the lowest, leading to low diversity indices. Diversity indices in A, M and B were very similar and relatively high due to the large OTU numbers (24, 19 and 23 OTUs, respectively; S3 Table) in addition to their high dominance (low evenness) levels (Table 3).

The rank abundance curves of A, M, B, and A+M+B non-ream (S2 Fig) are similar in shape; they have steep slopes, demonstrating a low evenness of OTUs, and long tails, indicating that the majority of the sequences belong to rare organisms represented by only a few reads [46]. HW, MW, A+M+B and Random showed rank abundance curves with shallower slopes, demonstrating higher evenness.

The results are very similar when diversity is analyzed only for *Trebouxia* (Table 4), with some differences in algal composition and diversity indices of A, M and B present. The apical zone of the laciniae (A) showed lower evenness and diversity, whereas the basal zone (B) showed a very high evenness and diversity.

**Table 4:** Diversity indices only for *Trebouxia*.

	HW	MW	A	M	B	A+M+B	A+M+B non-ream	Random
Richness (S) =	5	5	18	16	18	4	13	10
Margalef Index (Dmg) =	0.50	0.49	2.07	1.99	2.40	0.35	1.38	1.23
Shannon Index (H´) =	1.25	0.91	0.94	1.50	3.13	0.08	0.25	1.19
Simpson Index (D´) =	1.79	1.38	1.30	1.68	6.40	1.02	1.05	1.49
Evenness (J´) =	0.54	0.39	0.22	0.37	0.75	0.04	0.07	0.36

## Discussion

HTS approaches are providing unprecedented amounts of information to assess species diversity at diverse taxonomic levels and in diverse ecological settings. These techniques have been progressively applied in lichenological studies throughout the past few years; some authors have noted how the sample storage, the selection of primers and the application of different DNA extraction protocols can influence the final perception of species diversity and its patterns [18,47]. Furthermore, lichens represent peculiar microecosystems, whose insights regarding the associated microorganism diversity are still in the early stages [3,48–51]. Bacteria, fungi and basidiomycete yeast have recently been shown to interact in lichen symbioses, and their diversity and localization in the thalli have been investigated by both HTS, *in situ* hybridization and high-resolution microscopy [11,17,20]. Although the multiplicity of microalgal partners—the autotrophic partners of these symbioses—has been revealed for many years, it has not yet been sampled by the most recent techniques and remains underestimated.

Here, we carried out a set of 454 pyrosequencing analyses to ascertain in the most comprehensive way the diversity of symbiotic microalgae associated with lichen thalli. In doing this, we took particular care in developing a protocol to handle the lichen material and to amplify the DNA to confirm the co-occurrence of multiple microalgae and to test whether these are differentially localized in the thalli. The lichen *Ramalina farinacea* proved to be the best reference model for these experiments, as previous knowledge on co-occurring photobionts was available, and this lichen presents a thallus morphology suitable for establishing parallel assays [2,3,11].

The analyses of seven treatments from a single thallus (thallus A) of *Ramalina farinacea* detected a total of 31 OTUs, which were representative of four photobiont genera (*Trebouxia*, *Asterochloris*, *Vulcanochloris* and *Diplosphaera*; S3 Table). In addition to confirming the co-occurrence of *Trebouxia* sp. TR9 and *T*. *jamesii*, this result highly enlarges the spectrum of algal diversity previously known for *R*. *farinacea*.

Our experimental design aimed at differentiating the fraction of epithalline algae from those that are strictly intrathalline by analyzing the water washing samples, HW and MW. In HW and MW, we detected 8 and 6 OTUs, respectively, including *Trebouxia* sp. TR9 and *T*. *jamesii*, which were also recovered from treatments A, M and B of thallus A. These two *Trebouxia* either were derived from the inner part of the thallus, due to possible fragmentation during the washing procedures, or were detached from the superficial soredia. Due to the verified co-occurrence of both *Trebouxia* inside *R*. *farinacea* thalli [3], this was considered to be intrathalline algae. The OTUs exclusively detected in HW and MW (A54 and *Elliptochloris*) and those recovered both in the washing treatments and from the inner parts of thallus A (N1, *T*. *asymmetrica*, *T*. *cretacea*, *T*. *solaris*, A55, *Asterochloris*, and *Vulcanochloris*) as well as of thallus B (*T*. *asymmetrica*, *T*. *cretacea* and *T*. *solaris*) were considered to be the epithalline algal fraction.

The different proportions of *Trebouxia*, *Asterochloris* and additional green microalgae OTUs found in the apical (A), middle (M) and basal (B) zones of the thalli suggest a differential localization of these microalgae along the laciniae, which has already been proposed by García et al. [52] for *R*. *farinacea*. Similarly, a differential distribution of symbionts is known for bacterial communities in coral hosts [53]. In fruticose lichens, such as *R*. *farinacea*, the thallus parts selected as starting material may represent an important factor influencing the results; therefore, this material may be a key parameter to be considered in future experimental designs. Based on these results, it can be hypothesized that the differential distribution of the phycobionts may be potentially correlated with lichen morphogenesis. Several authors [54–56] have described diffuse or intercalary growth in the fruticose *Ramalina menziesii*, *R*. *usnea* and *Usnea longissima*. Unfortunately, such data are not available for *R*. *farinacea*. The presence of a discontinuous central cord formed at the apical zones in *R*. *farinacea* [57] along with the high relative abundance of *Trebouxia* OTUs in this part, as pointed out in this study, supports the apical growth type of this species. The variation in photobiont localization could be partially explained by the different environmental conditions [58] along the laciniae, which could suggest a succession of *Trebouxia* species inside the thallus, with pioneer algal communities in the apical zone, where they are exposed to more changeable environmental conditions. This hypothesis should be further confirmed by more detailed quantitative analyses and fluorescent *in situ* hybridization.

These results seem to be biased when the three thallus zones from thallus A are pooled (A+M+B) or when the samples are treated as the Random treatment from thallus B. In these three treatments—whether A+M+B was prepared with the reamplification strategy (A+M+B) or not (A+M+B non-ream)–the composition of microalgae is underestimated by the over-amplification of *Trebouxia* sp. TR9. Such unexpected discrepancy of OTU detection between the A+M+B; the Random; and the individual A, M and B treatments may be attributed to stochastic variations related to the PCR amplifications. The Random treatment, in particular, represents an example of a low coverage-level sample (Table 1), whereas sequence variants of middle and low frequency were not uncovered (1493 filtered reads) because the pyrosequencing was in a different run and included more MIDs (first run = 82 MIDs *vs* second run = 24 MIDs). The number of samples included on a plate can limit the total number of reads recovered per MID and should be taken into account when HTS assays are designed. Consequently, the overwhelming dominance of *Trebouxia* sp. TR9 significantly lowers the values of the diversity indices.

Studies on the microalgae diversity in lichens have been performed mainly by Sanger sequencing so far, but in using this procedure, only the primary phycobiont is detected [6,43]. In lichen thalli of *R*. *farinacea*, the two taxa, *Trebouxia* sp. TR9 and *T*. *jamesii*, were regularly recovered, and their co-occurrence was confirmed again in the present study. *Trebouxia* sp. TR9 was, however, the photobiont mostly sequenced by Sanger sequencing, suggesting that this method may limit the detection of further associated algae due to primer and/or amplification biases. The results obtained here from the 454 pyrosequencing analyses, in fact, showed that a multiplicity of microalgae are associated in the thalli, and their differential detection does depend on the thallus parts analyzed and the amplification protocol used. In this respect, customizations for the handling of material, the applied protocols and the bioinformatics pipelines for data analyses are required.

We experience that a preliminary RT-PCR amplification is necessary to determine the appropriate PCR cycle number to be applied when preparing the products for pyrosequencing. The PCR cycle number has to be optimized for each sample (the samples corresponding in this case to the different treatments; S2 Table) to avoid the over-amplification of the primary phycobiont and to detect the OTUs present at low and intermediate abundances in the lichen thalli. The consideration of samples from axenic algal cultures and a mock community is also used to assess the reliability of previously established bioinformatics pipelines. Although we assembled here the simplest artificial community, composed of only two taxa, the observed relative abundances of each *Trebouxia* species (86% *vs*. 14%) differed from those of the theoretical expectations (50% *vs*. 50%). This discrepancy between expected and observed relative abundances has also been detected in more complex mock communities, and it has been attributed to multiple factors, such as the (i) differential denaturation of the amplified fragments, (ii) binding efficiency of the sequencing primers, or (iii) accessibility of the target gene within genomes and/or the gene copy number in multi-template PCR [59,60]. It also notes that the data obtained for microalgal communities from the different treatments should be cautiously interpreted, as read abundance may be prone to PCR amplification biases and, therefore, may not reflect the true abundance of the taxa [61,62].

In this study, the 454 pyrosequencing of the GS Junior generated an enormous number of reads with intermediate lengths, which limited the base pair number of the genetic markers amplified. The selected ITS1-5.8S fragments represent a variable region used to identify microalgae, and the variable can be widely applied to discriminate *Trebouxia* species. However, when certain genera are detected, such as *Asterochloris*, species identification based only on the ITS1 region is not appropriate, and additional markers, e.g., actin, should be combined in the future [45].

In conclusion, our study provides evidence that a consistently planned handling of lichen material and its preparation for molecular HTS analyses is necessary to tackle and succeed in uncovering previously overlooked diversity of lichen-associated microorganism communities. To our knowledge, this is the first in-depth analysis of microalgal diversity through applied pyrosequencing approaches in lichens. Recently, Park et al. [19] have examined algal diversity in Antarctic lichens by HTS, but they showed the presence of only three *Trebouxia*, one *Asterochloris*, and two more green microalgae taxa in thalli of *Cladonia borealis*. The astonishing microalgal diversity that can be recovered in lichen thalli strengthens the concept of lichens as multispecies symbiotic ecosystems [8], where many rare or even still unknown species might be hosted and probably act as additional primary producers in the symbioses. However, the discovery of this diversity also raises further intriguing questions that should be considered in future lichen studies, including the following: a) How, when and why does the lichen mycobiont acquire many different microalgae? b) How are such diverse microalgae maintained in lichen thalli? c) Which selection mechanisms influence the phycobiont assemblages and their differential distribution in the thalli? and d) What are the ecological functions of the low-abundance microalgae in the thalli? The complementation of different technologies, such as ultrastructure microscopy, *ad hoc*-developed fluorescent *in situ* hybridization, culture isolations and transcriptomics need to be employed to thoroughly understand the diversity and the functions of microalgal communities in lichens. Such analyses will be relevant for ecological, physiological and evolutionary studies to explain how lichens adapt and outcompete also in the most extreme environments.

## Supporting information

S1 Fig *Trebouxia* haplotype networks. Statistical parsimony networks obtained for the ITS1-5.8S showing the relationships among haplotypes detected in the 26 *Trebouxia* OTU networks found in this study, including 23 sequences selected from the GenBank database. The size of the circles is proportional to the frequency of each haplotype in the total sample and is indicated with a number next to the circle. Each line in the network represents one mutational step; small white circles represent missing haplotypes that were not observed in the data. Each network is indicated by boxes and named by the *Trebouxia* species and OTU designation, following the same color code of the clades of Helms [31] in the phylogenetic tree of Fig 3. The color coding for the eight treatments is reported at the bottom of the figure.
(JPG)

S2 Fig Rank abundance graph of algal OTUs recovered from each treatment. The number of OTUs were ordered from most to least abundant on the X axis, and the relative abundance of each type (number of sequences) observed was plotted on the Y axis.
(JPG)

S1 Table Taxonomic identification of the algal cultures and the primary algae amplified from the seven treatments. (DOCX)

S2 Table Average Cycle threshold (Ct) values obtained for the RT-PCR I, RT-PCR II and RT-PCR in the algal cultures, the mock community and the eight PCR amplifications. (DOCX)

S3 Table Summary of presence/absence of *Trebouxia*, *Asterochloris*, and additional green microalgae and the total number of OTUs recovered for each treatment. (DOCX)

S4 Table Specific forward primers designed based on the nrITS DNA reference sequences of the OTUs obtained in the pyrosequencing assay. Only the primers that successfully amplified the targeted microalgae are reported.
(DOC)
