# Supplementary figures and images for: Unexpected associated microalgal diversity in the lichen Ramalina farinacea is uncovered by pyrosequencing analyses
Source: PLoS One. 2017 Apr 14;12(4):e0175091. doi: 10.1371/journal.pone.0175091 (PMC5392050; doi:10.1371/journal.pone.0175091)

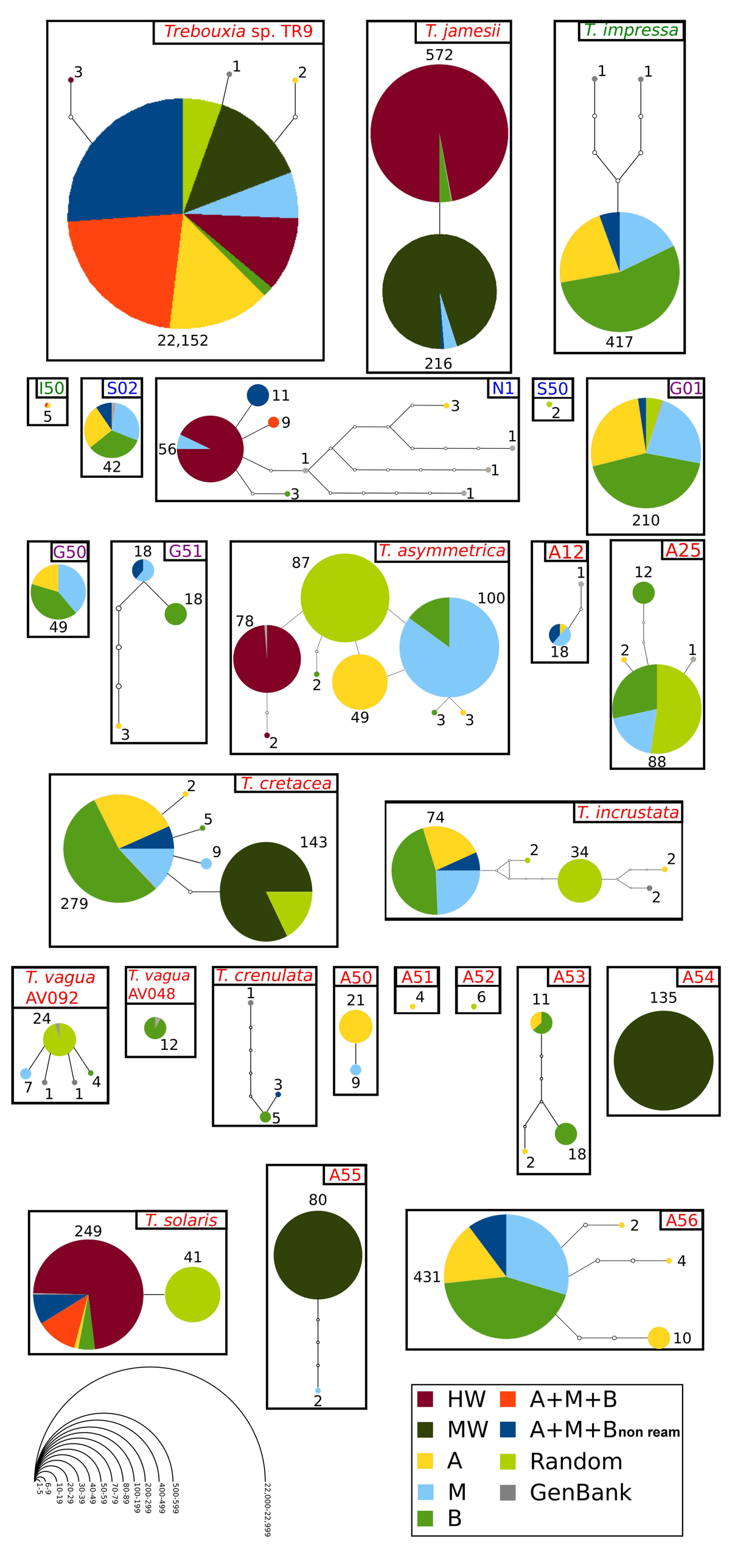

Supplement: S1 Fig — Statistical parsimony networks obtained for the ITS1-5.8S showing the relationships among haplotypes detected in the 26 Trebouxia OTU networks found in this study, including 23 sequences selected from the GenBank database. The size of the circles is proportional to the frequency of each haplotype in the total sample and is indicated with a number next to the circle. Each line in the network represents one mutational step; small white circles represent missing haplotypes that were not observed in the data. Each network is indicated by boxes and named by the Trebouxia species and OTU designation, following the same color code of the clades of Helms [31] in the phylogenetic tree of Fig 3. The color coding for the eight treatments is reported at the bottom of the figure. (JPG) [file pone.0175091.s001.jpg]

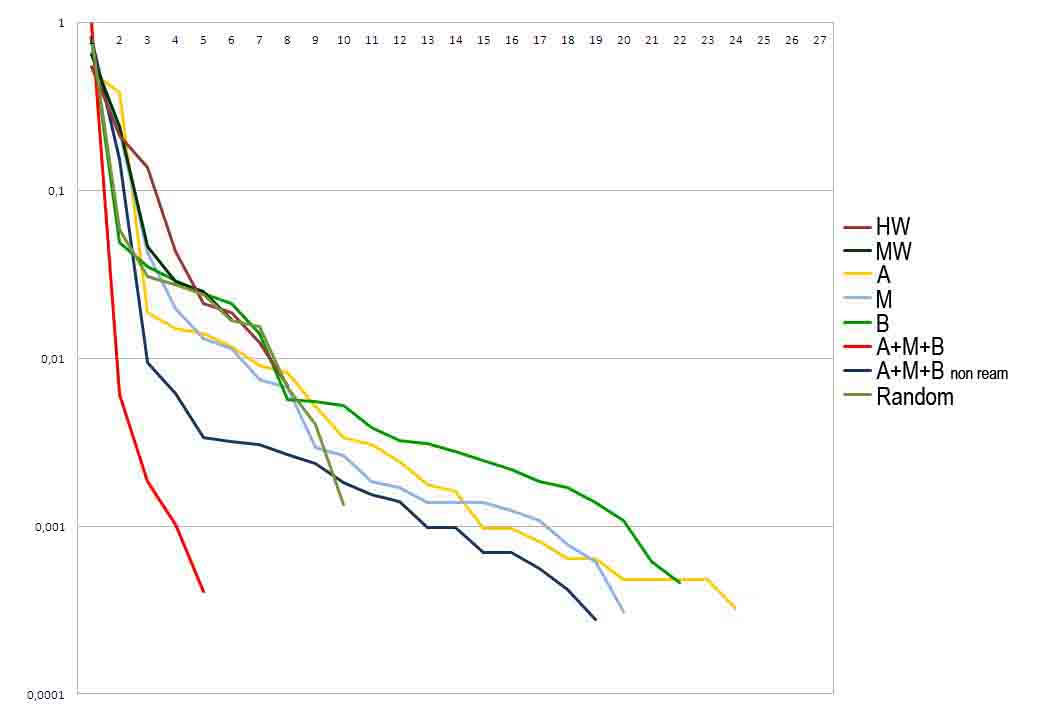

Supplement: S2 Fig — The number of OTUs were ordered from most to least abundant on the X axis, and the relative abundance of each type (number of sequences) observed was plotted on the Y axis. (JPG) [file pone.0175091.s002.jpg]
