# Supplementary material for: Unexpected associated microalgal diversity in the lichen Ramalina farinacea is uncovered by pyrosequencing analyses
Source: PLoS One. 2017 Apr 14;12(4):e0175091. doi: 10.1371/journal.pone.0175091 (PMC5392050; doi:10.1371/journal.pone.0175091)
Supplement: S1 Table — (DOCX) [file pone.0175091.s003.docx]

**S1 Table. Taxonomic identification of the algal cultures and the primary**

**algae amplified from the seven treatments.**

| Treatment name | **BLAST match (1)** | **GenBank number** | **BLAST match (2)** | **GenBank number** |
| --- | --- | --- | --- | --- |
| ***Trebouxia jamesii* SAG 2103** | *T*. *jamesii* voucher AV031 | KT819977 |  |  |
| ***T. asymmetrica* SAG 48.88** | *Trebouxia* Trinkaus 450 | AJ293784 |  |  |
| ***Trebouxia* sp*.*TR9** | *Trebouxia* sp*.* TR9 | KU716051 |  |  |
| **HW** | *Trebouxia* sp*.* TR9 | KU716051 | *Asterochloris mediterranea* | KT215311 |
| **MW** | *Trebouxia* sp*.* TR9 | KU716051 |  |  |
| **A** | *Trebouxia* sp*.* TR9 | KU716051 |  |  |
| **M** | *Trebouxia* sp*.* TR9 | KU716051 |  |  |
| **B** | *Trebouxia* sp*.* TR9 | KU716051 | *Asterochloris mediterranea* | KT215311 |
| **A+M+B** | *Trebouxia* sp*.* TR9 | KU716051 |  |  |
| **Random** | *Trebouxia* sp*.* TR9 | KU716051 |  |  |
