# Supplementary material for: Unexpected associated microalgal diversity in the lichen Ramalina farinacea is uncovered by pyrosequencing analyses
Source: PLoS One. 2017 Apr 14;12(4):e0175091. doi: 10.1371/journal.pone.0175091 (PMC5392050; doi:10.1371/journal.pone.0175091)
Supplement: S2 Table — (DOCX) [file pone.0175091.s004.docx]

**S2 Table.** **Average Cycle Threshold (Ct) values obtained for the RTPCR**

**I, RT-PCR II and RT-PCR in the algal cultures, the mock community and the eight PCR amplifications.**

| **DNA samples** | **Ct values**  **RT-PCR I** | **Ct values**  **RT-PCR II** | **Ct values**  **RT-PCR** |
| --- | --- | --- | --- |
| *Trebouxia jamesii* | - | - | 20 |
| *Trebouxia asymmetrica* | - | - | 23 |
| Mock community (*T. jamesii* / *T. asymmetrica*) | - | - | 21 |
| *Trebouxia* sp.TR9 | - | - | 30 |
| *Ramalina farinacea* apical zone (A) | 23 | 7 | - |
| *Ramalina farinacea* middle zone (M) | 25 | 6 | - |
| *Ramalina farinacea* basal zone (B) | 28 | 7 | - |
| *Ramalina farinacea* A+M+B | 21 | 6 | - |
| *Ramalina farinacea* A+M+B non ream | - | - | 26 |
| *Ramalina farinacea* Random | 20 | 10 | - |
| HW | 29 | 5 | - |
| HM | 29 | 5 | - |
