# Supplementary material for: Unexpected associated microalgal diversity in the lichen Ramalina farinacea is uncovered by pyrosequencing analyses
Source: PLoS One. 2017 Apr 14;12(4):e0175091. doi: 10.1371/journal.pone.0175091 (PMC5392050; doi:10.1371/journal.pone.0175091)
Supplement: S3 Table — (DOCX) [file pone.0175091.s005.docx]

**S3 Table. Summary of presence/absence of *Trebouxia*, *Asterochloris*, additional green microalgae and total number of OTUs recovered for each treatment.**

|  | ***Trebouxia*** | | | | | | | | | | | | | | | | | | | | | | | | | |
| --- | --- | --- | --- | --- | --- | --- | --- | --- | --- | --- | --- | --- | --- | --- | --- | --- | --- | --- | --- | --- | --- | --- | --- | --- | --- | --- |
|  | **Clade I** | | **Clade S** | | | **Clade G** | | | **Clade A** | | | | | | | | | | | | | | | | | |
|  | ***T. impressa*** | **I50** | **S02** | **N1** | **S50** | **G01** | **G50** | **G51** | ***Trebouxia* sp. TR9** | ***T. jamesii*** | ***T. asymmetrica*** | **A12** | **A25** | ***T. cretacea*** | ***T. incrustata*** | ***T. vagua* AV092** | ***T. vagua* A091** | ***T. crenulata*** | ***T. solaris*** | **A50** | **A51** | **A52** | **A53** | **A54** | **A55** | **A56** |
| **HW** | - | - | - | x | - | - | - | - | x | x | x | - | - | - | - | - | - | - | x | - | - | - | - | - | - | - |
| **MW** | - | - | - | - | - | - | - | - | x | x | - | - | - | x | - | - | - | - | - | - | - | - | - | x | x | - |
| **A** | x | x | x | x | - | x | x | x | x | - | x | x | x | x | x | - | - | - | x | x | x | - | x | - | - | x |
| **M** | x | - | x | x | - | x | x | x | x | x | x | x | x | x | - | x | - | - | - | x | - | - | - | - | x | x |
| **B** | x | - | x | x | - | x | x | x | x | x | x | - | x | x | x | x | x | x | x | - | - | - | x | - | - | x |
| **A+M+B** | - | x | - | x | - | - | - | - | x | - | - | - | - | - | - | - | - | - | x | - | - | - | - | - | - | - |
| **A+M+B non ream** | x | - | x | x | - | x | - | x | x | x | - | x | - | x | x | - | - | x | x | - | - | - | - | - | - | x |
| **Random** | - | - | - | - | x | x | - | - | x | - | x | - | x | x | x | x | - | - | x | - | - | x | - | - | - | - |

|  | ***Asterochloris* sp.** |
| --- | --- |
|  |  |
|  |  |
| **HW** | x |
| **MW** | x |
| **A** | x |
| **M** | x |
| **B** | x |
| **A+M+B** | x |
| **A+M+B non ream** | x |
| **Random** | - |

|  | **Additional algal diversity** | | | | | | | |
| --- | --- | --- | --- | --- | --- | --- | --- | --- |
|  | **OTU1** | ***Elliptochloris*** | **OTU3** | **Chlorophyta URa28** | **OTU5** | **Chlorophyta URa26** | ***Vulcanochloris*** | ***Diplosphaera*** |
| **HW** | - | x | - | - | - | - | x | - |
| **MW** | - | - | - | - | - | - | - | - |
| **A** | - | - | x | x | x | x | - | x |
| **M** | - | - | - | - | x | x | - | - |
| **B** | - | - | - | x | x | x | x | - |
| **A+M+B** | - | - | - | - | - | - | - | - |
| **A+M+B non ream** | x | - | - | x | x | x | - | x |
| **Random** | - | - | - | - | - | - | - | - |

+

|  | **Total number OTUs** |
| --- | --- |
|  |  |
|  |  |
| **HW** | 8 |
| **MW** | 6 |
| **A** | 24 |
| **M** | 19 |
| **B** | 23 |
| **A+M+B** | 5 |
| **A+M+B non ream** | 19 |
| **Random** | 10 |
