# Supplementary material for: Unexpected associated microalgal diversity in the lichen Ramalina farinacea is uncovered by pyrosequencing analyses
Source: PLoS One. 2017 Apr 14;12(4):e0175091. doi: 10.1371/journal.pone.0175091 (PMC5392050; doi:10.1371/journal.pone.0175091)
Supplement: S4 Table — Only the primers that successfully amplified the targeted microalgae are reported. (DOC) [file pone.0175091.s006.doc]

**S4 Table. Specific forward primers designed based on the nrITS DNA reference sequences of the OTUs obtained in the pyrosequencing assay**. Only the primers which successfully amplified the targeted microalgae are reported.

| **Code specific primer** | **Sequence specific primer (5’-3’)** |
| --- | --- |
| *Trebouxia* OTU A52 | TTCTGGAATCGGCAGGCTTTGTTGTTT |
| *Trebouxia solaris* | TTCTGGAGTAGGCAGGTTTCATTGTCT G |
| *Trebouxia* N1 | ACAAGCGGTAGCTCCCCTTGGGGAGTTG |
| *Trebouxia crenulata* | AACAGGCAAACTTTATTGTTTGCCCTTCAG |
| *Trebouxia* OTUA25 | GTAGGCAGATTTTACCGTTTGCCCTT |
| *Trebouxia asymmetrica* | CCGGCAAACTTTATTGTTTGCCCTTC |
| *Trebouxia jamesii* | TAGGCAGGCTTCACAGCCTGCCCT |
| *Trebouxia* sp. TR9 | TCTGACCTTCAGTTGCGCAGGTCAG |
| AD_OTU1 | CATTGTGCCAACCAACCGGTCAC |
| *Asterochloris* sp. | TGTTGGCCTGGCTGCCAAAGGTTCACCC |
